# Insights into the Cellular and Molecular Mechanisms That Govern the Fracture-Healing Process: A Narrative Review

**DOI:** 10.3390/jcm10163554

**Published:** 2021-08-12

**Authors:** Dionysios J. Papachristou, Stavros Georgopoulos, Peter V. Giannoudis, Elias Panagiotopoulos

**Affiliations:** 1 Unit of Bone and Soft Tissue Studies, Department of Anatomy-Histology-Embryology, School of Medicine, University of Patras, 26504 Patras, Greece; stavrosbuzuki@hotmail.gr; 2 Department of Pathology, School of Medicine, University of Pittsburgh, Pittsburgh, PA 15260, USA; 3 Academic Department of Trauma & Orthopaedics, School of Medicine, University of Leeds, Leeds LS1 3EX, UK; pgiannoudi@aol.com; 4 NIHR Leeds Biomedical Research Center, Chapel Allerton Hospital, Leeds LS7 4SA, UK; 5 Department of Orthopedic Surgery, University Hospital of Patras, 26504 Patras, Greece; ecpanagi@med.upatras.gr

**Keywords:** fracture-healing, bone remodeling, bone ossification, bone marrow adiposity, skeletal stem cells

## Abstract

Fracture-healing is a complex multi-stage process that usually progresses flawlessly, resulting in restoration of bone architecture and function. Regrettably, however, a considerable number of fractures fail to heal, resulting in delayed unions or non-unions. This may significantly impact several aspects of a patient’s life. Not surprisingly, in the past few years, a substantial amount of research and number of clinical studies have been designed, aiming at shedding light into the cellular and molecular mechanisms that regulate fracture-healing. Herein, we present the current knowledge on the pathobiology of the fracture-healing process. In addition, the role of skeletal cells and the impact of marrow adipose tissue on bone repair is discussed. Unveiling the pathogenetic mechanisms that govern the fracture-healing process may lead to the development of novel, smarter, and more effective therapeutic strategies for the treatment of fractures, especially of those with large bone defects.

## 1. Introduction

Fracture is a very frequent cause of morbidity with one third of males and one half of females being affected globally, at least once in their lifetime. Even though most fractures recover uneventfully, 2–11% of fractures, depending on which long bone has been traumatized, fail to unite, resulting in non-unions [1]. Importantly, this may strongly affect many aspects of the patient’s life leading to financial, family-related, and psychological issues. Indeed, it has been reported that the average direct cost of long bone non-union treatment is USD 11,333 (USA), USD 11,800 (Canada), and GBP 29,204 (UK) [2]. Not surprisingly, the socioeconomic burden imposed on the healthcare system by fractures has pushed the scientific community towards a synchronized effort to delve deeper and shed light upon the mechanisms of fracture repair to develop novel more effective therapeutic interventions for the successful management of bone injuries [3,4] For this to be achieved, a multidisciplinary approach employing different scientific specializations (i.e., medicine, biology, biomechanics, material sciences) is vital.

Fractures result in disruption of bone continuity, which in turn leads to changes in bone microenvironment and homeostasis. The process of fracture-healing aims at bridging the gap between the bone parts and at ensuring that injured bone will regain its pre-fracture structural and functional characteristics. To achieve this, a series of cellular and molecular events that recapitulate normal bone development take place. Two major types of fracture-healing processes have been recognized: (a) the primary (direct) healing, during which skeletal stem cells (SSC) directly differentiate into osteoblasts mirroring intramembranous bone formation; and (b) the secondary (indirect) healing that recapitulates mainly endochondral bone formation, since SSC transform to chondroblasts, which are gradually replaced by bone-forming osteoblasts [5]. 

In the present review we focus on secondary fracture-healing, since it is far more common than primary. We provide a brief overview of the distinct steps of bone fracture repair, as well as the current knowledge of the cellular and molecular events that underscore this phenomenon. In addition, the characteristics of the fracture mechano-environment and the role of bone marrow fat in the healing process are discussed.

## 2. Biology of Bone Formation

To better appreciate the molecular mechanism of fracture-healing, the principals of bone cellular and molecular biology will be briefly presented. 

### 2.1. The Skeletal Stem Cells

It is generally accepted that the mesenchymal stem cells (MSC) are adult bone marrow multipoint stem cells and as such they have the capacity of both self-renewal and differentiation towards specific, different cell types, namely osteoblasts, chondroblasts, fibroblasts and lipoblasts [6]. Casting doubt on this theory, the latest studies have suggested that the “MSC family” incorporates a huge variety of different cell types that have skeletogenetic, hematopoietic and tissue supporting capabilities [7]. Notably, not all MSC exert stem cell properties. Therefore, several scientists propose that the term Bone Marrow Stromal Cells (BMSC), should be used instead of MSCs [7]. SSC is the term applied for a specific subpopulation of BM stem cells that give genesis to osteoblasts and other skeletal cell types (i.e., chondroblasts) both in vivo and in vitro [8] and will be used in this review paper. SSCs are located beneath periosteum and endosteum, at the bone marrow sinusoids and around bone marrow adipocytes and have a specific phenotype (CD146/MCAM+, and PDGFa+, CD45−, CD31−) [8]. The role of SSC in fracture-healing is essential [9,10]. Notwithstanding, until today, it is not clear if the SSC that are recruited for fracture repair are the same with those involved in normal ossification processes. It is possible that trauma “reshapes” bone microenvironment, attracting a specific SSC subpopulation, distinct from those involved in normal bone development [11]. Of note, SSC involved in fracture-healing are in periosteum and BM; the involvement of circulating SSC in fracture repair is minimal [8]. Obviously, additional studies are required to identify and fully characterize SSC and thoroughly understand their behavior and role in fracture-healing.

### 2.2. Bone Remodeling: The Continuous, Well-Balanced Interplay between Bone-Forming and Bone-Resorbing Cells

**Figure 1:**
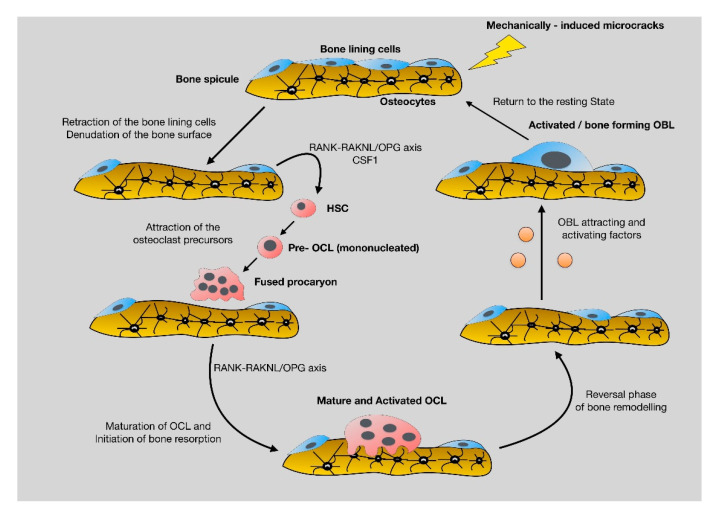
Schematic presentation of the cellular and molecular events that characterize bone remodeling. Briefly, microfractures that take place during every day normal activities activate the mechanoresponsive osteocytes, which in turn, through their cytoplasmic projections, send signals to bone lining cells that are retracted, leaving free space on bone surfaces for osteoclasts. Pro-osteoclasts are attracted to this denuded area and differentiate to mature and active multinucleate osteoclast that start bone resorption. During bone degradation osteoblast attracting molecules, such TGF beta are released, leading to diffraction and activation of osteoblasts that reach the resorbed area and start to synthesize new bone. The circle is repeated continuously, according to the applied mechanically forces. Disturbances in the well-balanced circle of bone remodeling result in bone pathologies, including inadequate fracture-healing.

In vertebrates, bone mass maintenance relies on the fine coordination between bone synthesis and bone resorption, two essential functions accomplished by highly specialized cells, the osteoblasts and osteoclasts, respectively. The crosstalk between these two cell types takes place throughout vertebrate life and are under the inspection of a delicate, yet complex process, named bone remodeling. Bone remodeling is controlled by a cassette of local (autocrine, and paracrine) and systemic (endocrine) signals and ensures the response of bone to mechanical and biochemical stimuli during physiological and pathological conditions, including traumatic injuries [12]. As such bone remodeling is crucial for bone homeostasis. Bone remodeling is characterized by a series of discrete, but not thoroughly defined, cellular and molecular events that are schematically presented in Figure 1. To appreciate the cellar and molecular events that underline bone remodeling the understanding of the major characteristics and functions of the cells of the family of bone-forming cells and the osteoclasts, is required. 

Osteoblasts are the bone-forming cells of the skeleton that originate from SSC. They have 2–4 nucleoli, very rich rough endoplasmic reticulum and Golgi apparatus, features that support their vigorous metabolic activity. Osteoblasts produce extracellular matrix that contains predominantly collagen type 1, as well as smaller proportions of other specialized proteins such as osteocalcin [13]. Notably, osteoblast differentiation and function is influenced by nearly all the common signal transduction pathways implicated in cell homeostasis [13]. The master regulators of osteoblastic differentiation are osterix and RUNX2; RUNX2 is an upstream osterix effector that regulate cre-recombinase for osteoblast-specific gene expression [14]. The signaling cascades implicated in osteoblast differentiation, proliferation and ultimately apoptosis are numerous and overlapping and include the mitogen-activated kinases (MAPK), the Wnt/β-catenin, the bone morphogenic proteins (BMP) and transforming growth factor-β (TGFβ), the hedgehog, the Notch, the insulin-like growth factor 1(IGF1), the fibroblast growth factor (FGF) and the calcium signal transduction pathways that regulate RUNX2 [15]. In addition to OBL the family of bone-forming cells also involve bone lining cells and osteocytes.

The bone lining cells (BLC) constitute a specialized subtype of OBL that line periosteal and endosteal surfaces. In fact, these cells are OBL that have entered a hibernation state, yet retain their ability to be activated and transformed to mature bone-forming OBL, if needed. From a histological standpoint BLC are spindle-shaped, with poorly developed rough endoplasmic reticulum and Golgi apparatus, features that fit well their low metabolic status The SNO cells (Spindle-shaped N cadherin positive, CD45 negative Osteoblasts) represent a subcategory of BLCs, which are the cardinal component of the BM osteoblastic niche participating in vital functions in vertebrates such as osteosynthesis, hematopoiesis, neoplastic diseases and bone response to trauma [16]. 

Osteocytes (OC) are the most abundant cells of the skeleton constituting the 90–95% of bone cells. In fact, OC are OBL that have been trapped in the ECM that they have produced. Since they are surrounded by dense bone, they have developed a sophisticated network of cytoplasmic projections that contain connexin-43 for the commutation with other bone cells and with the extracellular environment [17]. OC have the unique ability to sense even minute changes in their mechano-environment and to transforming mechanical cues to biochemical response through a process called mechanotransduction [18]. Even though it was previously believed that OC are terminally differentiated, post mitotic cells, it is now well appreciated that these cells can be potentially metabolically very active, participating in crucial processes of the vertebrates such as osteolysis (osteocytic osteolysis), bone remodeling and bone-healing after trauma [19]. Interestingly, OC have also the ability to affect the function of osteoblasts, both positively (via the secretion of anabolic mediators, such as Nitric Oxide and Prostaglandins E2) and negatively (via the production of the Wnt/β-catenin partway inhibitor, Sclerostin) [20]. 

In contrast to OBL, OCL stem from blood monocytes. They are large, multi-nucleated, metabolically active cells and as denoted by their name, their main function is degradation of bone and/or cartilage ECM. To start ECM degradation, OCL adhere tightly to bone surfaces via specific and specialized integrins, such as ανβ3 [13]. The osteolytic function of OCLs is facilitated by the secretion of acid proteinases, such as cathepsins K and matrix metalloproteases (MMP)-9 and -13 that degrade collagen fibers. Bone debris are removed chiefly by vacuolar translocation [14]. It is noteworthy that OCL differentiation and maturation is highly dependent upon OBL. Indeed, OBL produce and secret the ligand protein RANKL (receptor activator for nuclear factor kB-Ligand) that binds RANK, a receptor located on OCL cell membrane [21]. The formation of the RANK/RANKL complex is necessary for activation and differentiation of pre-OCL to mature, multinucleate and fully active OCL. In addition to RANKL, OBL also secrete Osteprotegerin (OPG), a decoy receptor that binds RANKL, preventing the RANK/RANKL complex formation and thus, OCL maturation and function [21]. Imbalances in the RANK/RANKL axis result in disturbances in bone homeostasis that lead to skeletal pathologies, such as osteoporosis, Paget’s disease, osteogenesis imperfecta and bone recovery after trauma. mCSF1 (macrophage colony stimulation factor-1) a molecule that, as with RANKL, is produced by osteoblasts and stromal cells, is also implicated in osteoclastogenesis, further underscoring the importance of the flawless OBL-OCL coupling in skeletal physiology and homeostasis.

During normal, everyday routine activities, mechanical forces applied to skeleton result in micro-cracks that are sensed primarily by osteocytes, the most abundant and, importantly, the most mechanosensitive cells of skeleton. Sensitized osteocytes transmit pre-apoptotic signals to specialized surface osteoblasts, the bone lining cells that undergo apoptosis leaving bone surface “uncovered”. This is sensed by pre-osteoclasts that will reach the denuded area, mature and eventually start bone degradation. When the function of osteoclasts is terminated, according to the needs of the organism, they undergo programmed cell death (apoptosis), leaving space for osteoblasts to synthesize new bone [13]. During bone resorption, quiescent growth factors (i.e., TBF-β) are activated, released from bone matrix and promote osteoblast activation, fueling the cycle of bone remodeling. Disturbances in the “coupling” between bone formation and bone destruction, results in bone-related pathologies, including metabolic diseases and problematic fracture-healing.

## 3. The Anabolic Phase of Fracture-Healing

Fractures are characterized by a complete or partial disruption in the continuity of bones that results in significant alterations at both the mechanical and the biochemical microenvironment of skeletal cells. The process of fracture-healing is a finely tuned interplay between different cell types of bone marrow that is mediated by endocrine and/or paracrine signaling pathways, which are stimulated largely by biomechanical cues [22]. Following trauma, the blood vessels of the Harvesian and Volkman channels, as well as the periosteal and BM blood vessels are destroyed. This results in depletion of oxygen and nutrient supplies, necrosis, acidosis and eventually hypoxia. Under hypoxic conditions, VEGF (vascular endothelial growth factor) is secreted from inflammatory cells, osteoblasts/stromal cells and hypertrophied chondrocytes. As a major endothelium-acting growth factor, VEGF promotes both angio- and vasculo-genesis at the sites of injury. Fracture induced hypoxia also activates HIF1-a (Hypoxia inducible factor 1-alpha) an upstream VEGF effector. Induction of the HIF-1a-VEGF cascade has a dual effect. First, it can trigger the up-regulation of genes related to the angiogenic cassette in the periphery of the disrupted blood vessels. This effect is mediated primarily the VEGF receptors, R1 (VEGFR1/FLT1) and R2 (VEGFR2/FLK1) [23] and leads to de-novo vascularization. Second, it can induce the shift of SSCs towards bone-forming osteoblasts [24]. Notably, HIF1-a can also facilitate the attraction and homing of SSC to fracture site, through activation of CLCX12-CXCR4 axis [25]. CLCX12 is released from the injured periosteum of the fracture bones, whereas CXCR4 is a surface receptor of SSC and bone marrow stromal cells. The aforementioned hypoxia-induced phenomena participate in the secondary (indirect) fracture-healing process that recapitulates endochondral ossification; the role of the hypoxia in primary fracture-healing that resembles intramembranous ossification, is less obvious.

**Figure 2:**
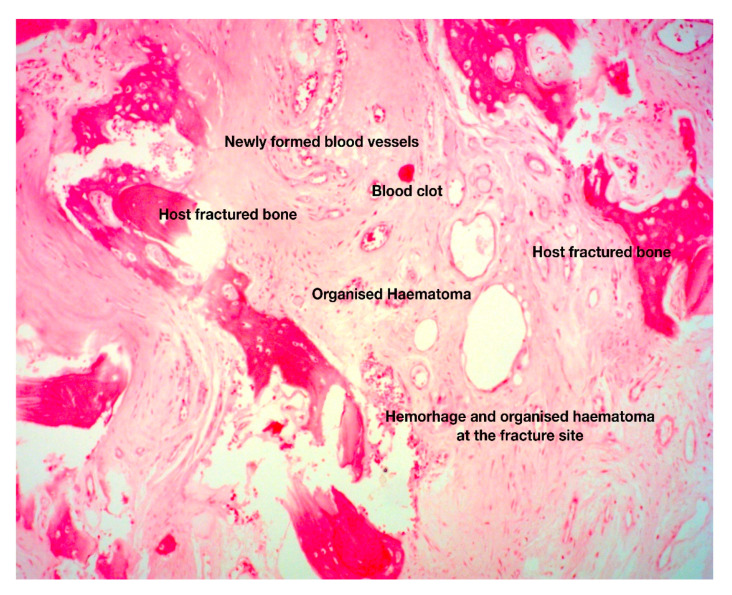
Histologic section of fracture site during the early, fibrous callus formation phase. The area between the fractured host bone is occupied by newly formed blood vessels in a background of loose connective tissue and organized hematoma. These are the histopathological hallmarks of the fibrovascular phase of fracture-healing (original magnification 5×).

Another event that takes place a few minutes after fracture is the development of hematoma and the formation of fibrin-rich blood clot (Figure 2). The role of clot in fracture-healing is multifaceted. Indeed, it causes hemostasis that is crucial for the survival of injured bones and potentially for the life of the injured individual [26]. It offers, to some extent, stabilization of the fractured area and serves as a scaffold for the formation of soft callus, as healing progresses. Blood clot secretes specific cytokines that trigger the initiation of the early (inflammatory) phase of fracture-healing [26]. Plasminogen is critical for indirect fracture-healing, since it can remove fibrin molecules via its catalytic properties [27].

During the first day of hematoma formation several types of inflammatory cells, namely lymphocytes, eosinophils, macrophages and neutrophils are attracted to the injured area. Among them, neutrophils are the most abundant and, importantly the most crucial, for the healing process. Indeed, neutrophils secrete cytokines, such as IL-6 and IL-10 that trigger the inflammatory response and chemotactic agents [i.e., C-X-C motif ligand 1 (CXCL1) and monocyte chemotactic protein-1] that activate perivascular monocytic lineage cells. In support of the aforementioned data, it has been relatively recently shown that significant reduction of the neutrophil population in the vicinity of the trauma is strongly correlated with prolonged fracture-healing course [28]. The role of cytokines in fracture is complex and obscure, since they can influence the healing process, both positively and negatively.

**Figure 3:**
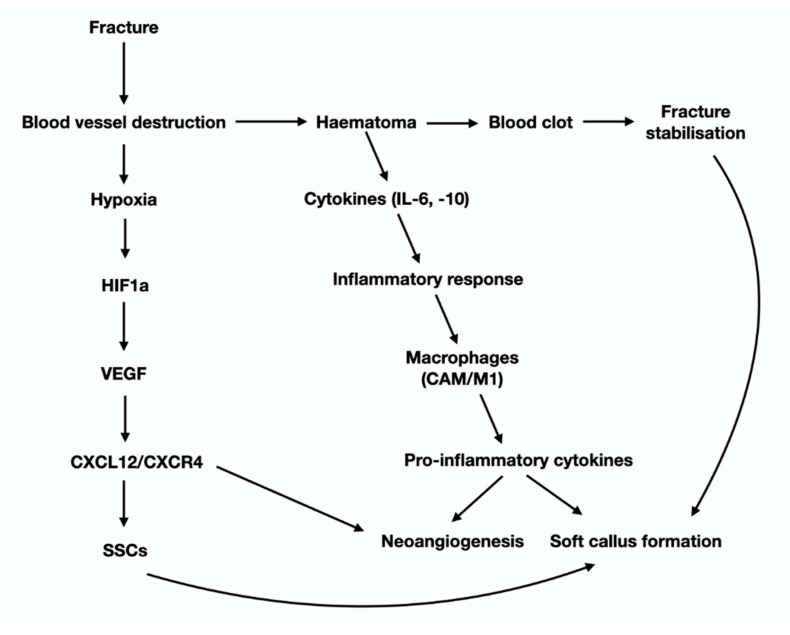
Graphical presentation of the cytokines and molecular signals that participate in the early steps of the anabolic phase of fracture hearing (see text for details).

Macrophages also participate in the inflammatory phase of fracture repair via secretion of the pro-inflammatory cytokines IL-1, IL-6, IL-10, TNF-a, FGFs, PDGF, TGF beta. Interleukins (especially IL-1, IL-6, IL-10), trigger IL-6 production from osteoblasts, which is responsible, among others, for angiogenesis and soft callus formation [29] (Figure 3). Native macrophages that reside in endosteum and periosteum, called “osteomacs”, promote soft callus synthesis through the production of MMPs (Matrix Metalloproteases) and BMPs (Bone Morphogenic Proteins) that facilitate the switch of SSC towards the osteoblast/chondroblasts lineage [30], affecting both the endochondral and the intramembranous ossification [31,32]. Except for their participation in the configuration of the pro-inflammatory microenvironment of the fracture site, macrophages also participate in the resolution of inflammation, by progressively removing the degraded fibrin clot and the cellular debris of hematoma phase [33], with phagocytosis. These anti-inflammatory macrophages are referred to as “alternatively activated macrophages” (AAM/M2), in contrast to the “classically activated macrophages” (CAM/M1) that possess pro-inflammatory characteristics. Cytokines, chemokines, and immunoregulatory cells crosstalk affects the balance between the polarization of CAM and AAM, subsequently influencing their function. AAM are under the impact of IL4 and IL13, whereas CAM are controlled by IFN-γ. Bone marrow-derived stromal cells and regulatory T cells also direct M2 cell polarization mainly through IDO (indoleamine 2,3-dioxygenase) and IL-10. AAM are highly phagocytic and exert their anti-inflammatory and tissue reshaping functions via specific cytokines (such as IL10) and other factors that endorse tissue re-vascularization [34].

**Figure 4:**
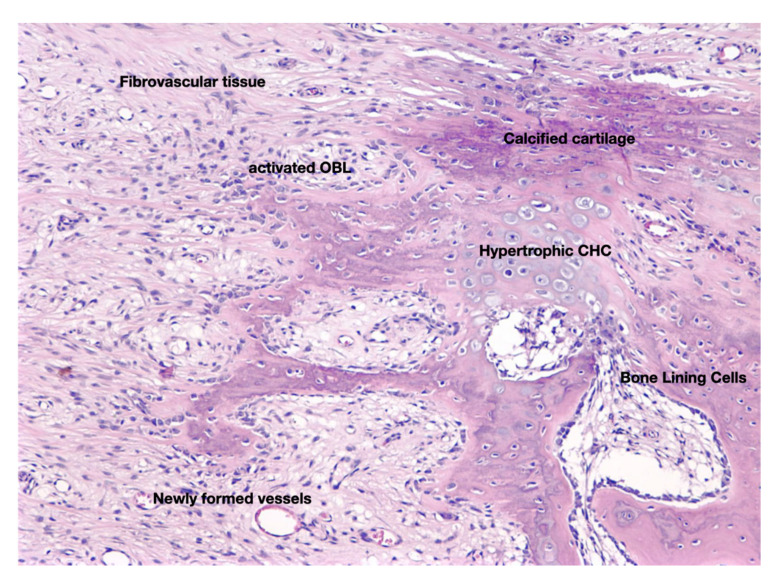
Histological section that depicts the cellular and molecular features of fibrocartilaginous callus which is formed during the anabolic phase of fracture-healing. Hypoxic conditions and mechanical stimulation determine whether SSC will follow the osteoblastic or the chondroblastic line of differentiation, giving genesis to bone and cartilage respectively, two cardinal elements of this phase of the fracture repair process. At the end of the anabolic phase the fractured bone parts are stabilized by hard (boney) callus (original magnification 5×).

**Figure 5:**
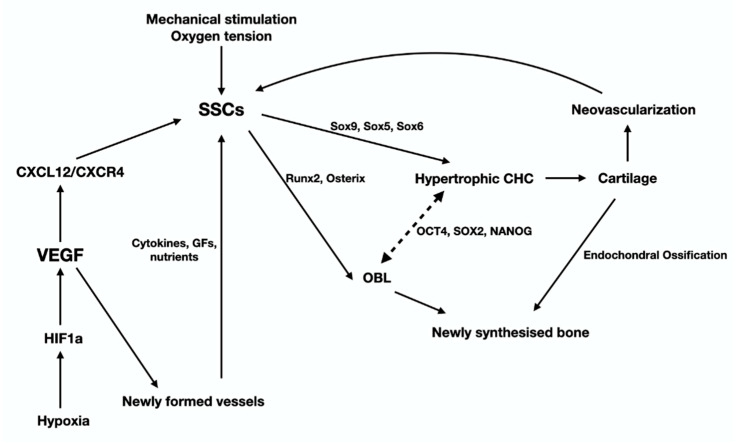
This graph illustrates the cells, signaling pathways and transcription factors that regulate the late stage of the anabolic phase of fracture-healing that leads to the synthesis of fibrocartilage and eventually bone, via a process the recapitulates endochondral ossification (see text for details).

Another effect of fracture is that it causes significant alterations to the mechano-environment of the injured bones. These alterations are sensed by OCTs, the most mechanosensitive cells of the skeleton, which in turn activate periosteal, endosteal and/or BMSC either directly through their cytoplasmic projection, or indirectly via paracrine stimuli. HIF1-a (via the CLCX12/CXCR4) facilitates SSC recruitment and homing and advances neovascularization. Activation of SSC and the subsequent revascularization of the ECM produced, launch the “fibrovascular phase” that follows the “inflammatory phase” of fracture-healing (Figure 4). The molecular mechanisms of revascularization have been presented earlier in this review paper. The fate of SSC at the fracture site, depends primarily on two parameters: the tension of oxygen and the level of mechanical stimulation. The cascade of events that are regulated by mechanical stimulation an oxygen tension is graphically presented in Figure 5. As mentioned previously, low oxygen tension favors the development of cartilage. Sox9 and, at lesser extent, other Sox family members (Sox 5 and 6) are the “master regulators” of chondroblastic differentiation. In fact, low stability and hence increased and persistent motion between the fragmented bone parts results in remarkable vascular depletion and curtailed oxygen tension that encourage chondrogenesis. On the other hand, adequate stability between the injured bones promotes osteogenesis, while restricting chondrogenesis, paralleling normal endochondral ossification. The switch of SSC towards osteoblast lineage is regulated by Runx2, the master regulator of osteoblastogenesis. Osterix, a downstream molecule of Runx2 is also highly involved in this anabolic process. It is worth mentioning that Runx2 is also expressed during the early and late (hypertrophic chondrocytes) stages of endochondral ossification. Nevertheless, Runx2, in contrast to SOX9, is not expressed during the biogenesis of the permanent hyaline cartilage of the articular joints. Histological analysis of the fracture site reveals the presence of chondrocytes from all the zones of cartilage maturation, i.e., the resting, hyperplastic, hypertrophic, and calcified cartilage. Notably, the role of hypertrophic chondrocytes is central in the process of fracture-healing. More specifically, they produce angiogenetic factors that promote a second neovascularization cycle, attracting osteoprogenitors and ostoblastogenetic factors such as Runx2, elements of the Wnt-β-catenin signaling axis and BMPs (bone morphogenic protein). BMP-5 and -6 have critical involvement in the differentiation and proliferation of chondrocytes, which is mediated by IGF1, JAK2, PKC, PTH, and IHH-PTHrP signal transduction pathways [35,36]. Up-regulation of the aforementioned osteoblastogenic factors and pathways is accompanied by down-regulation of the chondroblastogenic genes of the SOX family. Calcification of the hypertrophic chondrocytes that is accomplished mainly via BMP-related signals offers additional stability to the injured area. ECM of hypertrophied chondrocytes contains augmented levels of collagen type X and fibronectin that enable cartilage mineralization by calcium carbonate [37]. The molecular and spatial events that underline the cartilage-to-bone transition are still vague. The classic, rather dogmatic theory suggests that calcified hypertrophic chondrocytes are terminally differentiated, postmitotic cells that eventually undergo apoptosis; the calcified cartilage that remains is remodeled by the so-called “chondroclasts” to form marrow spaces. Howbeit, more recent studies have shown that hypertrophic chondrocytes are actually mitotically and metabolically agile cells, able to undergo trans-differentiation towards osteoblasts by Runx2 expression and the canonical Wnt/β-catenin pathway activation (Day et al., 2005; Hill et al., 2005/Hu) [38,39,40]. Interestingly, they also have the capacity to enter a stem-like state and acquire plasticity as defined by expression of the pluripotent transcription factors OCT4, SOX2 and NANOG [40]. As such, they can go through asymmetric cell division and either apoptose or reenter the cell cycle and follow the osteoblastic line of differentiation [40]. Indeed, hypertrophic chondrocytes display elevated expression of the osteoblastogenic factors Runx2, Wnt, osteocalcin, osteonectin and osterix; on the contrary, the levels of the chondrogenic markers Sox9, Col2α1 and Col10α1, are diminished [40]. The premature osteoid that is produced following the cartilage-to-bone transition is the hallmark of the fibrocartilaginous (or soft) callus and corroborate further stabilization of the fracture (Figure 3). As the healing process progresses, immature osteoid undergoes calcification and mature lamellar bone is produced, giving genesis to the hard (bony) callus. The anabolic phase of fracture repair is strongly impacted by biomechanical forces applied on the fractured area. Inadequate mechanical stimulation decreases osteoblastic differentiation and activation, whereas it boosts osteoclastic maturation and function, leading to inadequate fracture-healing [12]. On the other end of the spectrum, hypermobility and reduced stability between the fracture parts, results in the development of pseudoarthrosis that require additional surgical interventions. From a histopathological standpoint, pseudoarthroses are characterized by the presence of hyaline cartilage and synovium similar to those of normal articular cartilage. Even though hard callus offers sufficient stabilization of the fractured parts, it does not have the structural-biomechanical characteristics and the functional adequacy of a healthy bone. In order regain its’ normal properties, hard callus sustains a second cycle of resorption and trimming, which is mastered by osteoclasts. Degradation of the excess of bone/hard callus is the hallmark of the catabolic phase of fracture-healing.

## 4. The Catabolic Phase of Fracture-Healing

**Figure 6:**
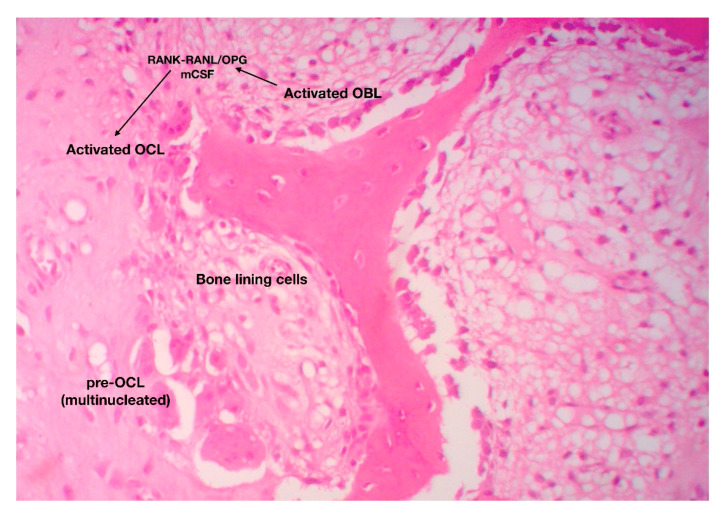
The catabolic phase of fracture-healing is characterized by reshaping of the hard (boney) callus. The RANK-RANKL axis has a central role in the differentiation, maturation and activation of bone-resorbing osteoclasts (original magnification 10×).

The catabolic phase of fracture-healing is accomplished by the bone-resorbing cells of the skeleton, the osteoclasts (OCL) (Figure 6). OCL are “surface cells” and as such, they must tightly attach bone surfaces, through specialized integrin (mainly ανβ3) adhesions, to exert their function. Cell membrane infoldings, rich in actin filaments, called ruffled border, are responsible for the attachment of OCL to bone surfaces and mark the area to be resorbed. Acid-secreting H+-ATPases [41] assist acid secretion to the resorption (Howship’s) lacunae, which is further enhanced by chloride-proton exchange and chloride channels [42,43]. OCL secret specific acid proteinases, namely cathepsins K and matrix metalloproteases (MMP)-9 and -13 that degrade collagen and other ECM constituents. Bone debris is removed primarily by vacuolar trafficking [14].

The function of OCL is under the direct and/or indirect control of endocrine/systemic (calcitonin, PTH) and local (paracrine) factors. It has been previously mentioned that OCL differentiation, proliferation and maturation relies upon the RANK-RANKL/OPG axis and m-CSF-dependent pathways [44].

OCL participates in several steps of fracture-healing. More specifically, during the early, inflammatory phase, osteoclastic activity is peaking due to the need for removal of excess debris and extracellular matrix from the wounded area. This process is mediated by neutrophil-derived pro-inflammatory cytokines such as TNF-a, IL-6, CCl-2, which recruit macrophages and activate signaling pathways that promote osteoclastic activity [44]. During indirect fracture-healing, OCL remove calcified cartilage from the injured area paving the way towards bone synthesis. Some authors use the term “chondroclasts” to describe these cartilage-destroying multinucleate giant cells; nonetheless it is now widely accepted that these cells share the same morphological and molecular feature with their bone-resorbing counterparts, the OCL. Finally, during the catabolic phase of fracture repair, OCL degrade and remove the excess of bony (hard) callus, reshape bone and eventually restore the pre-fracture architectural and functional features of the bones. Even though the anabolic phase of fracture-healing is completed in about 10–12 days, the catabolic phase lasts from 1 month to 2 years [45]. Interestingly, several studies and animal models have shown that inhibition of OCL has minor effect on cartilage but major effect on bone callus remodeling [46].

As with anabolic phase, the catabolic phase of fracture repair is also strongly influenced by mechanical stimulation. Indeed, reduced mechanical stimulation results in enhanced production of pro-inflammatory cytokines such as IL-6, TNF-α by osteoblasts, directly promoting osteoclast differentiation and maturation. In addition, low levels of mechanical loading lead in over-production of m-CSF and increase the RANKL/OPG ratio, favoring osteoclast activation and hence bone resorption [12].

## 5. The Role of Bone Marrow Fat in Fracture-Healing Process

Despite the prevailing view that adipose tissue is just a metabolically blunt, space occupying tissue, it is now well established that it exhibits significant metabolic activity and participates in vital physiological functions of the body, including response to endocrine and inflammatory stimuli. According to the classical concept, there are two types of adipose tissue: the white (WAT) and brown adipose tissue (BAT) that have distinct origin and different morphology and function.

From a micromorphological perspective, white adipocytes are spherical with centrally situated endocytoplasmic lipid droplet that pushes nucleus towards cell periphery. WAT regulates the balance between energy/nutritional availability and nutritional needs of the body, acting as a storehouse of excess calories. Moreover, it protects non-fatty tissues from toxic accumulation of nutrients [47]. WAT can communicate with other organs via endocrine mechanisms mediated by a variety fat-specific cytokines called lipokines [48].

Brown adipocytes are multilocular cells that possess a central role in the regulation of “thermogenesis”, a process ensuring the maintenance of basal body temperature after exposure to cold, in mammals [48]. In addition, BAT participates in the consumption of nutrients, and thus regulates body weight. From a molecular standpoint, BAT is characterized by an abundance of tightly packed mitochondria that contain the protein UCP1 (uncoupling protein 1) a potent regulator of thermogenesis. In addition to UCP1, other factors such as type 2 iodothyronine deiodinase (DIO2), UCP-1 transcription co-regulators PRDM16 and PGC1α, and the lipolysis modulator CIDEA, are part of the BAT-specific molecular cassette [49,50].

In recent years, a third category of adipose tissue that shares common features with both WAT and BAT, has been described. For obvious reasons, it is called “brite” or “beige” adipose tissue (BrAT). Brite adipocytes originate from Myf5- precursors, similarly to WAT; nevertheless, they are multivacuolated cells, rich in UCP1 expressing mitochondria. For this reason, it is believed that BrAT are morphologically and functionally closer to BAT. Notably, BrAT has greater thermogenic capacity than BAT, resulting in higher UCP1 levels [51].

WAT has low-grade chronic inflammatory properties, mediated by the pro-inflammatory cytokines tumor necrosis factor-α (TNFα), interleukine-6 (IL-6) and IL-1β. One of the main mechanisms leading to the synthesis and secretion of the aforementioned molecules is the binding of activated T-lymphocytes to macrophages. It is now well appreciated that inflammation affects anabolic and catabolic bone networks, and thus participate in the pathogenesis of metabolic, degenerative and neoplastic bone diseases. In particular, low-grade chronic inflammation inhibits the differentiation and proliferation of osteoblasts by suppressing important osteoinductive signaling cascades, such as Wnt/β-catecinin. Under the influence of the pro-inflammatory cytokines TNFα, IL6 and IL1β, proteins such as Dickkopf, Sclerostin, and secreted Frizzled-related proteins (sFRP) are activated, inhibiting the formation of Wnt–LRPs–FZD complex, which activates the osteoblastogenetic canonical Wnt/β-catenin pathway [52]. Furthermore, low-grade chronic inflammation suppresses additional pathways involved in osteoblast differentiation and activation, such as the IL6-JAK-STAT [(JUN N-terminal kinase)/(signal transducers and activators of transcription)], Mitogen-activated protein kinase (MAPK)—AP1, TNF—SMAD ubiquitylation regulatory factor (SMURF) 1/2) [53].

Low-grade chronic inflammation has also significant impact on the regulation of osteoclast function. More specifically, activated T-lymphocytes promote the synthesis of RANKL and prevent the production of OPG by osteoblasts, resulting in osteoclastic activation and maturation [54]. Notably, in skeleton, bone marrow adipocytes can induce osteoclastogenesis via RANK-RANKL-dependent and -independent mechanisms [55]. Additionally, the inflammatory ecosystem induces the synthesis of MCP1 (monocyte chemoattractant protein 1), a cytokine involved in the activation of several osteoclast-related signaling pathways, such as the JAK-STAT [55]. The effect of bone marrow BAT and WAT on bone metabolism has not been thoroughly investigated. Nonetheless, ample evidence indicates that the presence of BrAT and/or the conversion of WAT to BAT, have an anabolic effect on bones, which can be attributed, at least in part, to cytokines such as IL-1 and to modifications of sympathetic nervous system [56]. Very recent studies have shown that HDL deficiency is associated with reduced bone mass and decreased expression of specific BAT-related genes, suggesting a mechanistic link between HDL, bone marrow fat and bone metabolism [57,58].

## 6. Adipogenesis, Osteogenesis and Fracture-Healing

A rich body of clinical and research data suggest a close relationship between bone and fat metabolism. In fact, it has been documented that perturbations in lipid metabolic pathways, both local and systemic, have a strong impact on bone cells, resulting in numerous pathologies, such as osteoporosis and osteoarthritis. However, the molecular mechanisms that govern the bone-lipid connection remain insufficiently elucidated.

Noteworthy, mounting body of evidence over the past few years has pointed out that bone marrow adipose tissue greatly influences bone homeostasis and function. As a matter of fact, lipoblasts and osteoblasts represent the two sides of the same coin and thus, increased levels of one cell type are associated with decreased levels of the other. In line with that, increased marrow adiposity is followed by reduced bone mass, a phenomenon that is governed by a series of complex cellular and molecular events. LBL secrete factors such as chemerin, resistin, visfatin, leptin, adiponectin and omentin-1 that affect SSC program enhancing adipogenesis, while impeding osteoblastogenesis [59]. Others suggest that these molecules exert a direct action on hematopoietic stem cells HSC promoting osteoclastogenesis. In the same vein, bone marrow adipocytes synthesize RANKL, further contributing to OCL differentiation and maturation [59]. The augmented levels of inflammatory cytokines and free fatty acid from BM adipocytes enhance the lipogenic shift of SSC and osteoclastogenesis, affecting the susceptibility of bones to fractures, and their ability for fracture repair.

The tight connection between bone homeostasis and bone marrow adiposity is further reinforced by recent studies having investigated the role of HDL in the configuration of BM microenvironment. These studies have shown that HDL deficiency results in elevated BM adiposity and reduced bone mass, an effect that is mainly attributed to suppressed osteoblastogenesis, since osteoclast number and function remain unaffected [14,58].

Since bone marrow adiposity affects bone marrow microenvironment and skeletal biology, it could be speculated that it also affects the susceptibility of bone to fractures as well as its ability for uneventful healing. In fact, there are several research findings that support this speculation. Elevated BM adiposity is associated with the development of a pro-inflammatory microenvironment, which as mentioned previously, promotes lipo- and osteoclastogenesis resulting in reduced bone mass and hence increased susceptibility of bones to fractures. In addition, pathological conditions (i.e., diabetes type II, ageing, osteoporosis, HDL deficiency, metabolic syndrome) characterized by increased marrow adiposity and by a switch towards WAT, further increase the pro-inflammatory milieu of BM and hence, the capability of bone to recover from fractures, for all the reasons that have been addressed earlier in this paper.

Unraveling the molecular interface between bone and fat, it is likely to lead to the development of novel pharmaceutical agents (such as HDL-directed molecules) that could modify bone marrow microenvironment and accelerate fracture-healing progress.

## 7. Concluding Remarks

The past few years big steps have been made towards the comprehension of the mechanisms of fracture-healing. Regrettably however, the molecular, spatial and time-dependent phenomena that rule fracture repair have not been fully illuminated yet.

Not surprisingly, a considerable number of studies focus on the role of mechanical stimulation on the fracture-healing process, since mechanical forces promote osteoblastic activation and hence bone synthesis, while, on the hand, reduce osteoclast activation and function. The role of SSCs in the fracture-healing process warrants further investigation. Regrettably, it holds true that the exact location and the molecular features of SSC have not been identified till date. In addition, little is known as regards whether the SSC involved in skeleton repair are the same with those implicated in normal ossification processes. The considerable plasticity of SSC is a fertile field of research, since SSC-related manipulations may have anabolic impact on bone remodeling offering novel, and more importantly, efficient therapeutic solutions for the treatment of large fracture defects. Full characterization of SSC is also essential. Identification of specific markers may be useful to clarify which category of SSC (periosteal, endosteal, BM) orchestrates the fracture-healing process. Advanced molecular, cellular and imaging approaches on carefully selected animal models will considerably expand our knowledge towards this direction.

The role of bone marrow microenvironment also warrants further investigation. In fact, understanding the function bone marrow niches (osteoblastic, perivascular), as wells as, the role of bone marrow adipose tissue in the progression of fracture-healing might offer addition therapeutic solutions in the treatment of fractures.

Fracture-healing is physiological, yet extremely complex process that depends on the well-orchestrated collaboration of numerous different parameters (cellular, biochemical, biomechanical etc.). Importantly, even though bone-healing progresses flawlessly in most of the cases, a considerable proportion of fractures ends in delayed unions or non-unions, conditions that significantly affect patient quality of life. On that account, the demand for collaboration between research groups with different scientific background (medicine, biology, material sciences, pharmacy, biophysics) and industry is pressing. Indeed, the production of novel smart implants with the application of artificial intelligence, nanotechnology and advanced imaging that could sense, monitor and modulate/accelerate bone regeneration progress, constitutes on the most promising avenues towards the development of novel, personalized and more effective treatments of large bone defects and non-unions.

## Abbreviations

AAM: Alternatively activated macrophages, BAT: Brown adipose tissue, BLC: Bone lining cells, BM: Bone marrow, BMP: Bone morphogenic proteins, BMSC: Bone marrow stromal cells, BrAT: Beige adipose tissue, CAM: Classically activated macrophages, CD: Cluster of differentiation, CXCR: C-X-C chemokine receptor, ECM: Extracellular matrix, ESTIM: Electromagnetic field therapy, FGF: Fibroblast growth factor, FRP: Frizzled-related proteins, HDL: High Density Lipoproteins, HIF1-a: Hypoxia inducible factor 1-alpha, IDO: indoleamine 2,3-dioxygenase, IFN: Interferon, IGF1: Insulin-like growth factor 1, IHH: Indian hedgehog, PTHrP: Parathyroid Hormone related Peptide, IL: Interleukin, JAK: Janus kinase, LIPUS: Low intensity pulsed ultrasound, m-CSF: macrophage colony-stimulating factor, MAPK: Mitogen-activated kinases, MCAM: Melanoma cell adhesion molecule, MCP1: Monocyte chemoattractant protein 1, MMP: matrix metalloproteases, MSC: Mesenchymal stem cells, OBL: Osteoblasts, OC: Osteocytes, OCL: Osteoclast, OPG: Osteprotegerin, PDGFa: Platelet derived growth factor alpha, PKC: Protein kinase C, PTH: Parathyroid hormone, RANKL: Receptor activator for nuclear factor kB-Ligand, RUNX2: Runt-related transcription factor 2, SNO cells: Spindle-shaped N cadherin positive cells, SSC: Skeletal stem cells, TGF-β: Transforming growth factor beta, TNF-a: Tumor necrosis factor alpha, UCP1: Uncoupling protein 1,VEGF: Vascular endothelial growth factor, WAT: White adipose tissue, Wnt: Wingless-related integration site.
